# Advanced diffusion MRI and image texture analysis detect widespread brain structural differences between relapsing-remitting and secondary progressive multiple sclerosis

**DOI:** 10.3389/fnhum.2022.944908

**Published:** 2022-08-12

**Authors:** Olayinka Oladosu, Wei-Qiao Liu, Lenora Brown, Bruce G. Pike, Luanne M. Metz, Yunyan Zhang

**Affiliations:** ^1^Department of Neuroscience, Faculty of Graduate Studies, University of Calgary, Calgary, AB, Canada; ^2^Hotchkiss Brain Institute, University of Calgary, Calgary, AB, Canada; ^3^Department of Clinical Neurosciences, Cumming School of Medicine, University of Calgary, Calgary, AB, Canada; ^4^Department of Radiology, Cumming School of Medicine, University of Calgary, Calgary, AB, Canada

**Keywords:** multiple sclerosis, chronic active lesions, single-shell high angular resolution diffusion imaging, diffusion tensor imaging, along-tract statistics, phase congruency

## Abstract

**Introduction:**

Disease development in multiple sclerosis (MS) causes dramatic structural changes, but the exact changing patterns are unclear. Our objective is to investigate the differences in brain structure locally and spatially between relapsing-remitting MS (RRMS) and its advanced form, secondary progressive MS (SPMS), through advanced analysis of diffusion magnetic resonance imaging (MRI) and image texture.

**Methods:**

A total of 20 patients with RRMS and nine patients with SPMS from two datasets underwent 3T anatomical and diffusion tensor imaging (DTI). The DTI was harmonized, augmented, and then modeled, which generated six voxel- and sub-voxel-scale measures. Texture analysis focused on T2 and FLAIR MRI, which produced two phase-based measures, namely, phase congruency and weighted mean phase. Data analysis was 3-fold, i.e., histogram analysis of whole-brain normal appearing white matter (NAWM); region of interest (ROI) analysis of NAWM and lesions within three critical white matter tracts, namely, corpus callosum, corticospinal tract, and optic radiation; and along-tract statistics. Furthermore, by calculating the z-score of core-rim pathology within lesions based on diffusion measures, we developed a novel method to define chronic active lesions and compared them between cohorts.

**Results:**

Histogram features from diffusion and all but one texture measure differentiated between RRMS and SPMS. Within-tract ROI analysis detected cohort differences in both NAWM and lesions of the corpus callosum body in three measures of neurite orientation and anisotropy. Along-tract statistics detected cohort differences from multiple measures, particularly lesion extent, which increased significantly in SPMS in posterior corpus callosum and optic radiations. The number of chronic active lesions were also significantly higher (by 5–20% over z-scores 0.5 and 1.0) in SPMS than RRMS based on diffusion anisotropy, neurite content, and diameter.

**Conclusion:**

Advanced diffusion MRI and texture analysis may be promising approaches for thorough understanding of brain structural changes from RRMS to SPMS, thereby providing new insight into disease development mechanisms in MS.

## Introduction

Multiple sclerosis (MS) is a common disabling disease of the central nervous system characterized by inflammatory demyelination and neurodegeneration (Bagnato et al., 2020). Most patients start with a relapsing-remitting form (RRMS), but 60–70% of them progress to a secondary-progressive phenotype (SPMS) within 20–25 years of disease onset (Dutta and Trapp, 2014). This will lead to a critical shift clinically from transient symptoms to lasting disability with little functional recovery (Dutta and Trapp, 2014; Ontaneda, 2019). Various studies using magnetic resonance imaging (MRI) suggest the role of tissue pathology in the transition between these phenotypes in MS; however, the exact mechanisms are still unclear (Reich et al., 2018; Ontaneda, 2019). Thorough understanding of tissue changes underlying the structural and functional differences between RRMS and SPMS is essential, as that will help identify what and where to examine; ultimately, it will improve both our diagnosis and treatment evaluation capabilities.

Focal lesions remain to be a hallmark of MS pathology, but “invisible” abnormalities are shown to play an increasingly critical role in patient function (Vavasour et al., 2017; Filippi et al., 2018; Yu et al., 2019). Based on advanced MRI, including diffusion tensor imaging (DTI), high angular resolution diffusion imaging (HARDI), and myelin water imaging, studies of brain normal-appearing white matter (NAWM) in MS have found reduced measures in neurite density, dispersion, and myelin compared with healthy controls (De Santis et al., 2017; Vavasour et al., 2017; Spano et al., 2018; Rahmanzadeh et al., 2021). Texture analysis is another candidate measure of tissue microstructure achieved by assessing the characteristic relationships between adjacent voxels. On the one hand, recent evidence has also revealed extensive texture abnormalities in the NAWM of MS (Loizou et al., 2020). On the other hand, consequences of MS lesions also depend on their location in the nervous system (Bates et al., 2003). Based on DTI, white matter tractography, and magnetization transfer ratio, studies of major brain white matter tracts such as corpus callosum and corticospinal tracts have shown that patient dysfunction can be attributed to a single critical lesion impacting on myelin and axonal integrity (Reich et al., 2008; Tovar-Moll et al., 2015; Sechi et al., 2019; Ngamsombat et al., 2020). As such, regions of interest (ROIs) studies within white matter tracts may reveal important “hotspots” associated with disease evolution. Furthermore, changes distant from focal lesions are common in MS due to Wallerian degeneration (Klistorner et al., 2015). Therefore, diffusion MRI-enabled along-tract analyses are necessary for understanding both lesion and non-lesion pathology. Currently, there are studies related to specific aspects of the pathological spectrum, but they are not necessarily integrated as a whole (Reich et al., 2008; Harrison et al., 2011; Tovar-Moll et al., 2015; Ngamsombat et al., 2020).

Recent studies also suggest the importance of chronic active lesions to disease progression in MS (Dutta and Trapp, 2014; Absinta et al., 2019; Bagnato et al., 2020). While lesion development is often connected with clinical relapses in RRMS, many lesions in SPMS are chronic and smoldering, causing occult disease progression without signs of evident relapse (Dutta and Trapp, 2014; Reich et al., 2018). Histologically, chronic active lesions are characterized by inactive hypocellular demyelinated cores and actively inflammatory demyelinating rims (Dutta and Trapp, 2014). Characterizing the nature and extent of such chronic active lesions *in vivo* has become a critical priority to improve healthcare in MS; however, the availability of methods is limited (Klistorner et al., 2018; Bagnato et al., 2020). Current research has been relying on susceptibility-based imaging methods (Absinta et al., 2018), which define chronic active lesions as having isointense cores and hypointense rims (rim-positive) (Chawla et al., 2018; Filippi et al., 2019). The presence of more rim-positive lesions is associated with earlier disabilities in MS, and the persistence of paramagnetic rims from acute lesions suggests remyelination failure (Absinta et al., 2019). Nonetheless, susceptibility imaging is still under development and there is no evidence showing the ability of these methods to identify other pathologies such as axonal injury that is critical for MS progression.

This study aims to identify new quantitative methods for an integrated analysis of brain pathological changes in RRMS and SPMS and compare how and where they are different. The procedures will focus on novel analyses of diffusion MRI and image texture in clinical MRI.

## Materials and methods

### Sample

This study used brain MRI scans of 29 subjects with MS (all females), including 20 RRMS and 9 SPMS from two datasets as part of an ongoing clinical study (REB14-1926). Established criteria were followed in all diagnoses of MS (Polman et al., 2011), RRMS (Lublin et al., 2014), and SPMS (Lublin and Reingold, 1996). The first dataset (dataset1) included 10 patients with RRMS and nine patients with SPMS recruited for a study assessing corpus callosum function. The second dataset (dataset2) included 10 patients with RRMS as a convenience sample from a clinical trial of domperidone as a myelin repair agent (ClinicalTrials.gov Identifier: NCT02493049). For the latter, participants needed to have at least one gadolinium-enhancing lesion in brain MRI, but the current patients were ineligible and therefore did not continue in the trial. Both studies were approved by the institutional research ethics board. Written informed consent was obtained from all participants.

### Imaging protocol

Images of 3T anatomical and diffusion brain MRI were obtained from each dataset using a research-dedicated scanner (Discovery MR750; GE Healthcare, Milwaukee, USA). The imaging protocol included T1-weighted MRI acquired with a 1 mm isotropic, magnetization-prepared, fast-spoiled gradient echo sequence using 6.7–8.0 ms repetition time (TR), and 2.9–3.0 ms echo time (TE). T2-weighted MRI was acquired with a spin-echo sequence using TR1/TR2 = 6,000/5,600 ms and TE1/TE2 = 84/100 ms; matrix = 256 x 256/512 x 512; field of view (FOV) = 24 x 24/22 x 22 cm; and slice thickness = 3 mm. FLAIR MRI was obtained with a spin-echo inversion recovery sequence using TR1/TR2 = 7,000/6,000 ms and TE1/TE2 = 127/127 ms; matrix = 512 x 512; and FOV = 24 x 24 cm. Diffusion MRI was acquired with a spin-echo echo-planar sequence using TR1/TR2 = 8,000 ms and TE1/TE2 = 84/61 ms; matrix = 120 x 120; FOV = 24 x 24 cm; slice thickness=3/2 mm, 5 b0, with 23 b = 800 s/mm^2^ directions for Dataset1, and 3 b0, 45 b = 1,000 s/mm^2^ directions, and three reverse phase-encoded b0 for Dataset2.

### Diffusion MRI processing and analysis

#### Preprocessing

Image preprocessing for diffusion MRI involved several steps, which were essentially the same for dataset1 and dataset2 except the step used in susceptibility distortion correction due to the lack of reverse phase-encoded b0 data in dataset1. Briefly, the diffusion MRI scans were denoised, corrected for Gibbs ringing, and then bias corrected as reported previously (Veraart et al., 2016a,b; Cordero-Grande et al., 2019; Tournier et al., 2019; Oladosu et al., 2021). Eddy current and susceptibility distortion corrections were completed using the FSL eddy method (Andersson and Sotiropoulos, 2016; Andersson et al., 2016). The latter involved a tool called topup, where dataset1 was not compatible initially due to acquisition confounders as noted above. To compensate, we inverted the signal intensity of T1-w MRI from Dataset1 and rigidly transformed it to the diffusion space. The corresponding b0 volumes were averaged and nonlinearly registered (ANTs SyN) to the processed T1-w MRI in an x-axis constrained transformation to calculate susceptibility distortion (Avants et al., 2008; Huang et al., 2008). The distortions were transformed afterwards to a topup-like output format in FSL for correction (Andersson et al., 2003). Dataset2 was processed for susceptibility distortion correction using topup directly (Andersson et al., 2003). For both datasets, the corrected average b0 volumes were then rigidly registered (FSL epireg) to the corresponding T1-w MRI per patient for further processing (Jenkinson and Smith, 2001; Jenkinson et al., 2002). Next, diffusion images from the two datasets were harmonized for angular resolution by resampling, and for voxel-wise imaging characteristics by using the linear Rotationally Invariant Spherical Harmonics method based on 8 patients with RRMS (Descoteaux et al., 2007; Mirzaalian et al., 2018; Billah et al., 2019; Cetin Karayumak et al., 2019). These published accounts indicated that the harmonization approaches were valid if the b value differences between datasets fell between the range of 500 and 1,500 s/mm^2^.

To further clarify the suitability of the aforementioned harmonization methods, we calculated the variance and signal-to-noise ratio (SNR) of white matter ROIs and compared them between datasets based on harmonized data. Eight ROIs each with a size of 6 x 6 pixels were drawn in the corpus callosum, forceps minor, and forceps major tracts per subject, per examined diffusion measure. The SNR was evaluated relative to the standard deviation of the cerebrospinal fluid of the brain because the calculated maps were masked, which made the background of the maps all zeros. Subsequently, to enable HARDI analysis, new diffusion MRI data at b = 2,000 s/mm^2^ were predicted for both datasets based on their corresponding b = 1,000 s/mm^2^ data using an in-house deep learning algorithm (Murray et al., 2022).

#### Diffusion metrics calculation

**Figure 1:**
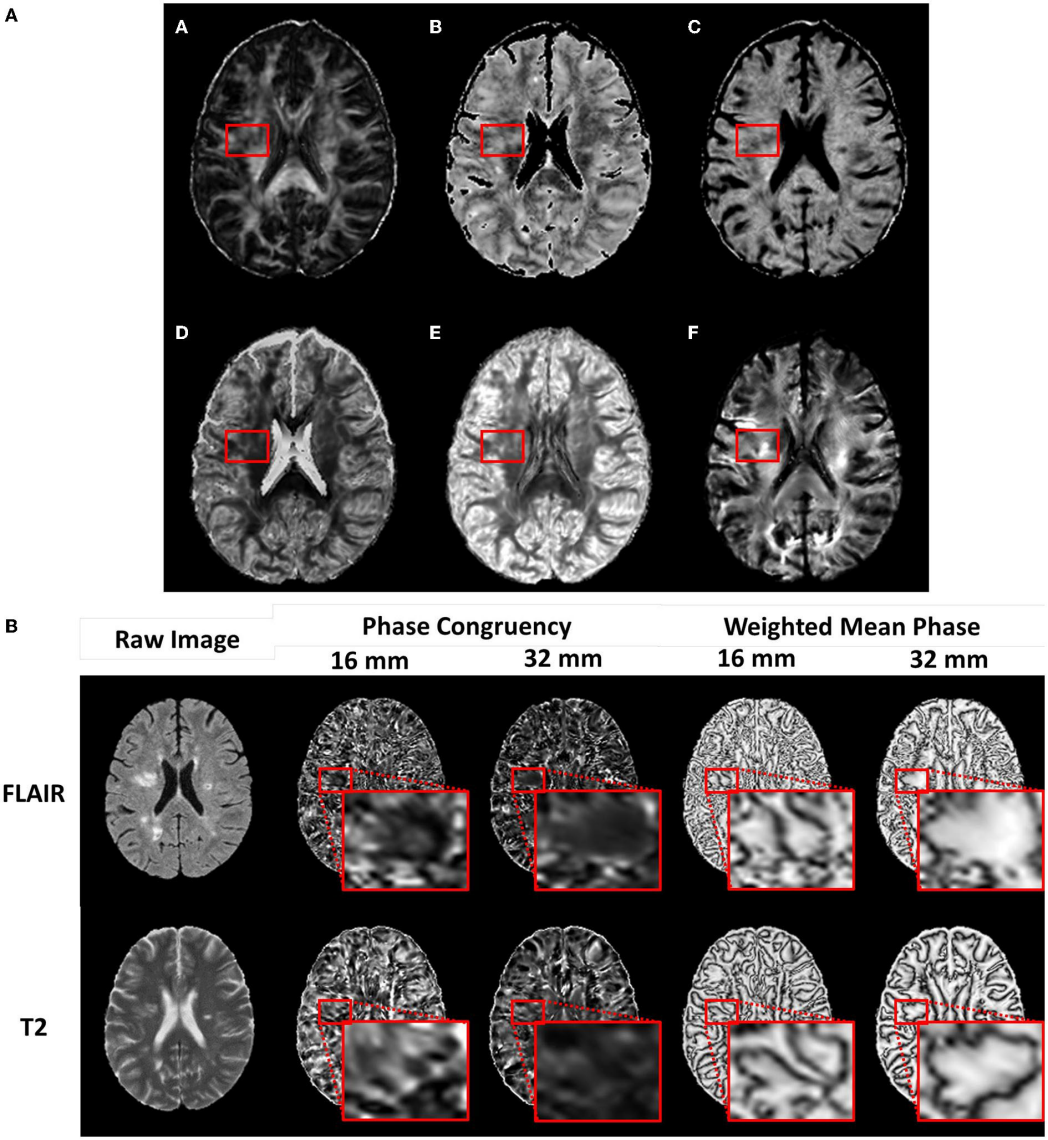
Example diffusion and phase congruency texture maps. **(A)** Diffusion MRI of a) fractional anisotropy, b) axonal diameter, c) intracellular volume fraction, d) Orientation Dispersion Index, e) Orientation Distribution Function energy f) Apparent Fiber Density, and **(B)** phase congruency of (top row) FLAIR MRI and (bottom row) T2 MRI.

Fractional anisotropy (FA) was obtained from DTI in FSL. HARDI analysis applied the ActiveAx method implemented in the accelerated microstructure imaging with convex optimization (AMICO) for crossing fibers (AMICOx) to model axonal diameter and intracellular volume fraction (ICVF), and neurite orientation distribution and density imaging (NODDI) in AMICO to calculate orientation dispersion (Alexander et al., 2010; Auria et al., 2015; Daducci et al., 2015). The apparent fiber density (AFD) was obtained using the fiber orientation distribution function (fODF), and ODF energy, a measure of orientational complexity, was obtained from the diffusion ODF computed by q-ball imaging reconstruction (Tournier et al., 2004; Descoteaux et al., 2007; Raffelt et al., 2012). All measures were transformed to the common MNI-152 coordinates for an analysis based on T1-w MRI nonlinear MNI transformation with the ANTs SyN method (Figure 1A).

#### Diffusion tractography

**Figure 2:**
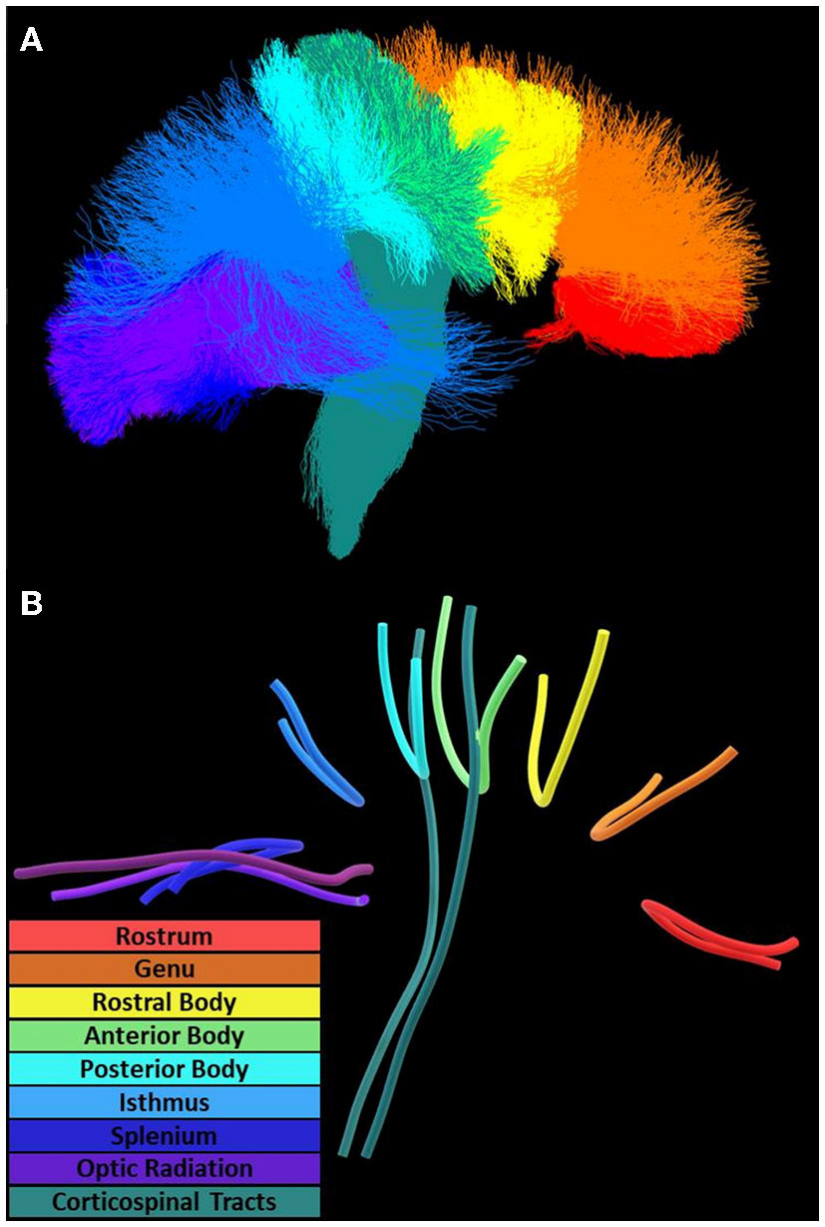
Diffusion tractography and tract geometries. Along-tract analysis of the corpus callosum, optic radiation, and corticospinal tracts utilized **(A)** tractography oriented posterior (left) to anterior (right) and **(B)** mean tract geometries for within-tract sampling.

The white matter fODF was calculated based on constrained spherical deconvolution using the b = 1,000 s/mm^2^ data alone. The resulting orientation distribution was nonlinearly transformed and reoriented to MNI-152 space using diffusion to T1 rigid and T1 to MNI nonlinear transformations (Tournier et al., 2004). The peaks of the fODF were calculated and input into a software known as TractSeg to obtain tracts and tract-ending segmentations (Wasserthal et al., 2018, 2019). Tract orientation mappings were then calculated and tractography generated through probabilistic tracking using iFOD2 and a dilation factor of 2 (Figure 2A) (Tournier et al., 2010). The corpus callosum was partitioned into seven segments according to the Witelson scheme based on locations of cortical intercepts (Witelson, 1989). The corticospinal tracts and optic radiations were also segmented bihemispherically.

### Texture analysis with phase congruency

Texture analysis was done for T2-weighted (T2-w) and FLAIR MRI using a 3D method called phase congruency. It was a frequency-based calculation approach and was shown to be insensitive to signal intensity differences between images (Kovesi, 1999). The same image preprocessing pipelines were applied to the anatomical images from dataset1 and dataset2. Essentially, the T1-w, T2-w, and FLAIR MRI were all preprocessed by Gibbs ringing correction, N4 bias field correction, and ANTs template-based brain extraction (Tustison et al., 2010; Kellner et al., 2016). Medial alignment of T1-w MRI was applied and that involved applying a rotation and translation procedure calculated from a rigid body registration to the MNI-152 T1 reference. T2-w and FLAIR MRI were then rigidly linearly transformed to the same dimensions as T1-w MRI. In addition, T2-w and FLAIR MRI were further processed with contrast-limited adaptive histogram equalization (Zuiderveld, 1994) (scikit-image v0.18.3) to enhance feature visibility thereby reducing the potential impact of the slightly different imaging protocols used in acquisitions.

Texture calculation produced two metrics, namely, phase congruency, reflecting edge strength based on the alignment of phases, and weighted mean phase, reflecting edge sharpness (Figure 1B) (Kovesi, 2003; Ferrari et al., 2011). Optimal calculation of these metrics required fine-tuning of several parameters (Table 1). Weighting adjustment for frequency spread used a sigmoid function with the inflection point (cutOff) set at 0.5 and degree of inflection (gain) set at 10.0. Filter bandwidths were regulated by


(1) $$\begin{array}{r} \frac{\sigma }{f_{0}}=0.55, \end{array}$$

by controlling the filter standard deviations (σ) relative to their central frequencies (*f*_0_). Central frequencies were separated by a factor (M) to obtain even spectral coverage. M was empirically determined as given by


(2) $$\begin{array}{r} M=\;{\frac{\sigma }{f_{0}}}^{\log\left(\frac{\pi }{20}\right)}. \end{array}$$

By default, the median of the highest frequency filter was used to characterize noise with the noise threshold set at two standard deviations. Filters were uniformly oriented on a sphere to balance orientational coverage according to a diffusion MRI gradient scheme of 23 directions conveniently available in this study. The number of filter scales was determined by


(3) $$n_{scale}=\;\left\lceil \frac{\log\frac{f_{\max}}{f_{\min}}}{\log M}\right\rceil +n\;|\;f_{\max}=\frac{1}{\lambda_{\min}},\;f_{\min}=\frac{1}{\lambda_{\max}},$$

including a heuristically determined *n*=2 additional filters to ensure uniform sensitivity at low frequencies. The frequency domain is bounded by the minimum and maximum wavelengths (λ). For images with a 1 mm^3^ voxel resolution, λ_min_ was set at 2 mm and λ_max_ at 16 and 32 mm to allow an analysis of frequencies around the spatial scale of most lesions observed in MS. Increased phase congruency and decreased weighted mean phase suggest increased signal complexity.

**Table 1:** Definition and impact of phase congruency parameters.

**Parameter**	**Symbol**	**Meaning**	**Impact**
**Spectral coverage**			
Minimum wavelength	**λ_min_**	Determines the highest frequency in analysis	Determines the smallest scale features for which patterns are detected
Maximum wavelength	**λ_max_**	Determines the lowest frequency in analysis	Determines the largest scale features for which patterns are detected
Number of scales	nScale	Number of filters to define a filter bank covering the frequency range (λ_min_, λ_max_)	Defines a set of filters for sensitivity across all feature frequencies
**Spectral sensitivity**			
Sigma	σ	Standard deviation of a single filter around its central frequency (*f*_0_).	Regulates the frequency coverage of a single filter
Multiple	M	The factor separating *f*_0_ of successive filters in a filter bank.	Together with σ, regulates how features at each frequency are relatively weighted
**Angular resolution**			
Number of orientations	nOrient	The number of filter banks positioned in 3D to detect features in multiple orientations	Provides representation of features at all orientations
**Frequency spread penalty**			
Cut-Off	cutOff	The inflection point of a sigma curve differentiating high and low frequency spread	Weights features with different orientations based on the complexity of their frequency makeup
Gain	g	The sharpness of a sigma curve in contrasting high over low frequency spread.	

### Outcome generation

This analysis focused on four scales of abnormalities that are highly associated with disease development in MS, namely, whole-brain NAWM, tract-based ROIs, along-tract changes, and chronic active lesions.

#### Whole-brain white matter

A histogram analysis method based on 256 bins was used to assess whole-brain NAWM. The procedure started with brain tissue segmentation with an open-source software (FSL FAST) using T1-w MRI. Focal MS lesions were segmented based on T1 and FLAIR MRI as reported previously (Oladosu et al., 2021). Eventually, this step provided three-dimensional ROIs for individual lesions. These lesion ROIs were dilated by one voxel and then subtracted from the FSL-segmented brain white matter to obtain the NAWM for each patient (Zhang et al., 2001). For each investigated imaging measure, the 50th (p50), 75th (p75), and 95th (p95) percentile, and histogram peak were collected.

#### NAWM and lesion regions within major white matter tracts

The ICBM-DTI-81 atlas of size 1 mm helped identify major brain white matter tracks, including three corpus callosum segments (Genu, Body, Splenium), bihemispheric corticospinal tracts, and optic radiation tracts (Mori et al., 2004; Wakana et al., 2007; Hua et al., 2008). The union of ROIs from the corticospinal tract, cerebral peduncle, posterior limb of the internal capsule, and superior corona radiata formed the overall corticospinal tract. NAWM and lesions were defined by intersecting whole-brain NAWM and lesion ROIs, respectively, with each tract.

#### Along-tract statistics

Tractometry was applied to tractography to obtain measurements for all investigated diffusion metrics at 100 points along the mean geometries of each investigated tract using distance map correspondence (Figure 2B) (Maddah et al., 2008; Wasserthal et al., 2020). Lesion maps were also averaged at each point along a tract giving a measure of the extent of local lesions (lesion extent), with values of 1 (one) indicating complete lesion coverage at that node. Coordinates and measurements at each point in corresponding mean geometries were further aligned across patients using diffusion profile realignment based on FA (St-Jean et al., 2019).

#### Chronic lesion activity analysis

**Figure 3:**
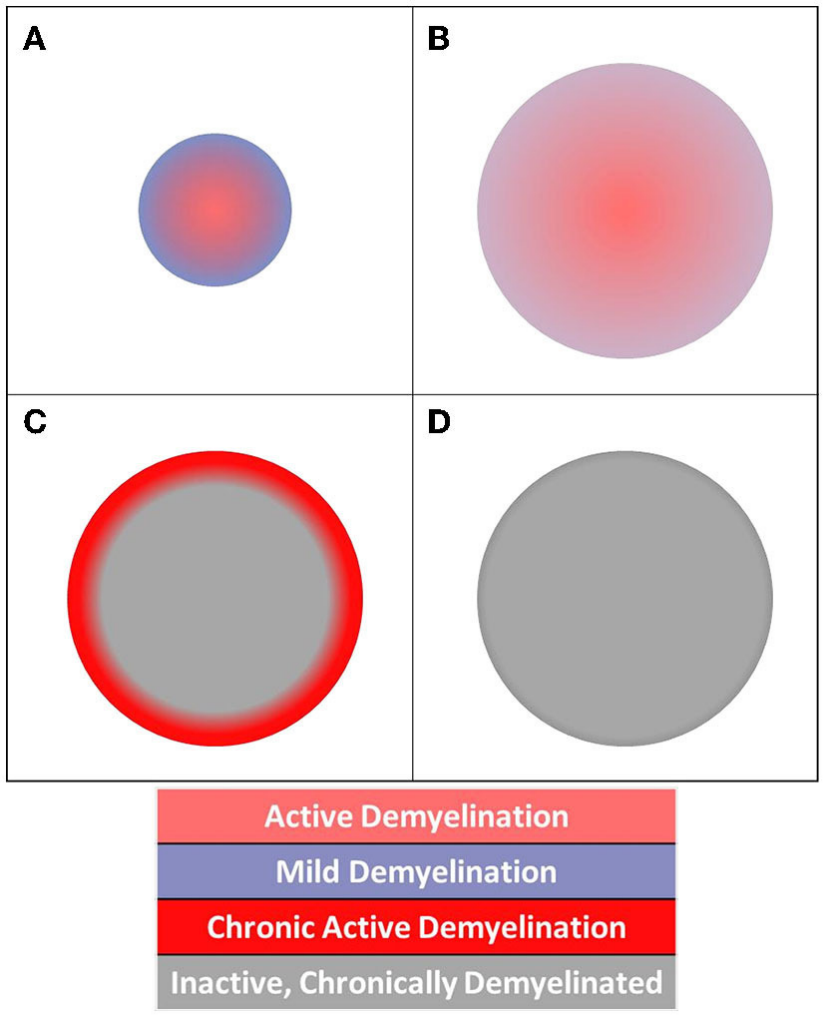
Diagram of acute and chronic lesion activity. Different patterns of pathology are visible in **(A)** acute lesion, **(B)** slowly expanding lesion, **(C)** chronic active lesion, and **(D)** chronic inactive lesion structures.

We proposed a schema to understand the activity of chronic MS lesions based on their core-rim dichotomy (Figure 3). Lesions were assigned a z-score based on the relationship between lesion core and rim pathology.


(4) $$\begin{array}{r} Z_{lesion}=\frac{\mu_{Core}-\mu_{Rim}}{\sigma_{Rim}} \end{array}$$

Lesion cores were defined by 26-connectivity erosion of lesion masks. Subtracting the core voxels from full-lesion ROIs produced single-voxel-thick rim ROIs for each lesion (Oladosu et al., 2021). Lesions without definable cores and rims were excluded in this step of the analysis. With this schema, chronic active lesions would present with z-scores>0 (or < 0 based on the investigated measure) highlighting greater core damage.

### Statistical analysis

All analyses focused on cohort differences between RRMS and SPMS. Histogram features for each measure were compared using ANOVA and then *post-hoc* Tukey correction for multiple comparisons. For tract-based ROI analyses, measures in tracts were compared using ANOVA for a combined NAWM and lesion analysis and then a linear mixed-effect model with subject as a random effect, including Tukey correction for pairwise comparisons to understand individual group differences. Tract-based means were compared using ANOVA, and along-tract variations were compared with a mixed-effect model and corrected for multiple comparisons using permutation testing. All models included subject age as a covariate. The sex factor was not controlled because all subjects were female. Disease duration was not included as a covariate because it was expected to be different between cohorts given the nature of SPMS being a continuum of RRMS. Analysis of multiple features and tracts was addressed with Benjamini-Hochberg correction. Two-sample comparisons used Student's *t*-tests, with *p* < 0.05 considered significant. For chronic active lesion analysis, the overall lesion percentages, and average counts of lesions per patient at multiple thresholds were graphed and tabulated.

## Results

### Sample characteristics

The mean (standard deviation) age of the participants was 46.9 (11.5) years, which was 40.7 (9.3) years for RRMS and 58.2 (8.9) years for SPMS subjects. The disease duration of the whole cohort was 15.5 (11.8) years, and it was 8.6 (6.5) years for RRMS and 29.3 (8.4) years for SPMS participants. Furthermore, the overall expanded disability status scale (EDSS) score was 3.3 (2.4), which was 1.9 (1.1) and 6.5 (0.5) for RRMS and SPMS subjects, respectively. In total, we identified 1,026 brain white matter lesions, 1 to 111 per subject. Patients with SPMS had an average of 48.56 lesions and patients with RRMS had an average of 29.45 lesions. Among the 1,026 lesions, 275 had core-shell analysis (SPMS: 12.67/pt, RRMS: 8.05/pt). In total, six diffusion and eight phase congruency measures were analyzed. Diameter, ODI, ODF energy, and FLAIR WMP showed higher values in regions of greater pathology such as those in SPMS vs. RRMS, while AFD, FA, ICVF, and T2 WMP showed the opposite trend, being lower in SPMS than RRMS. In addition, after harmonization of diffusion MRI, there was no significant difference (*p* > 0.05) in either variance or SNR of white matter ROIs between dataset1 and dataset2 for any calculated diffusion metrics (Supplementary Table 1).

### Histogram statistics

**Figure 4:**
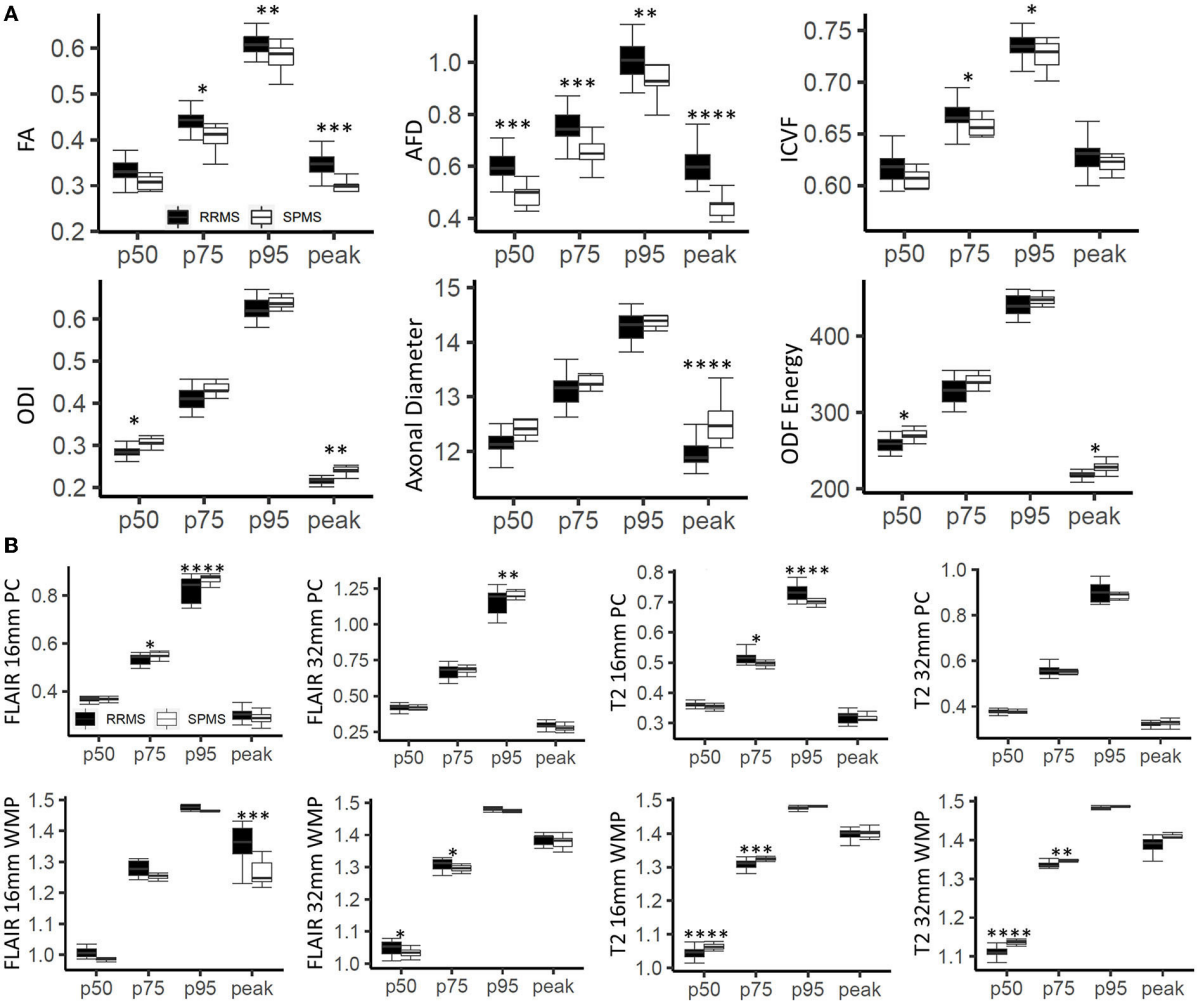
Histogram-based outcomes by cohort. **(A)** Demonstrates results for diffusion measures, and **(B)** for texture measures from phase congruency. The stars indicate *post-hoc* significance: *p < 0.05, **p < 0.01, ***p < 0.001, ****p < 0.0001. The boxes plot the median, interquartile range (IQR), and 1.5*IQR.

Analysis of variance showed that histogram features differed significantly between RRMS and SPMS for all diffusion and texture measures except T2 (32 mm) phase congruency (Figure 4). Diffusion-based AFD (*p* < 0.0001) differentiated cohorts across all four histogram features (*p* < 0.01); FA differentiated cohorts in all features but p50. Remaining diffusion measures showed cohort difference in only two histogram features (*p* < 0.05) except for diameter, which only showed significance in histogram peak (*p* < 0.0001). T2 and FLAIR (16 mm) phase congruency showed significance in both p75 and p95 (*p* < 0.05), while FLAIR (32 mm) and T2 (16 and 32 mm) were significant at p50 and p95 (*p* < 0.05) in differentiating cohorts.

### ROI-based tract-wise analysis

**Figure 5:**
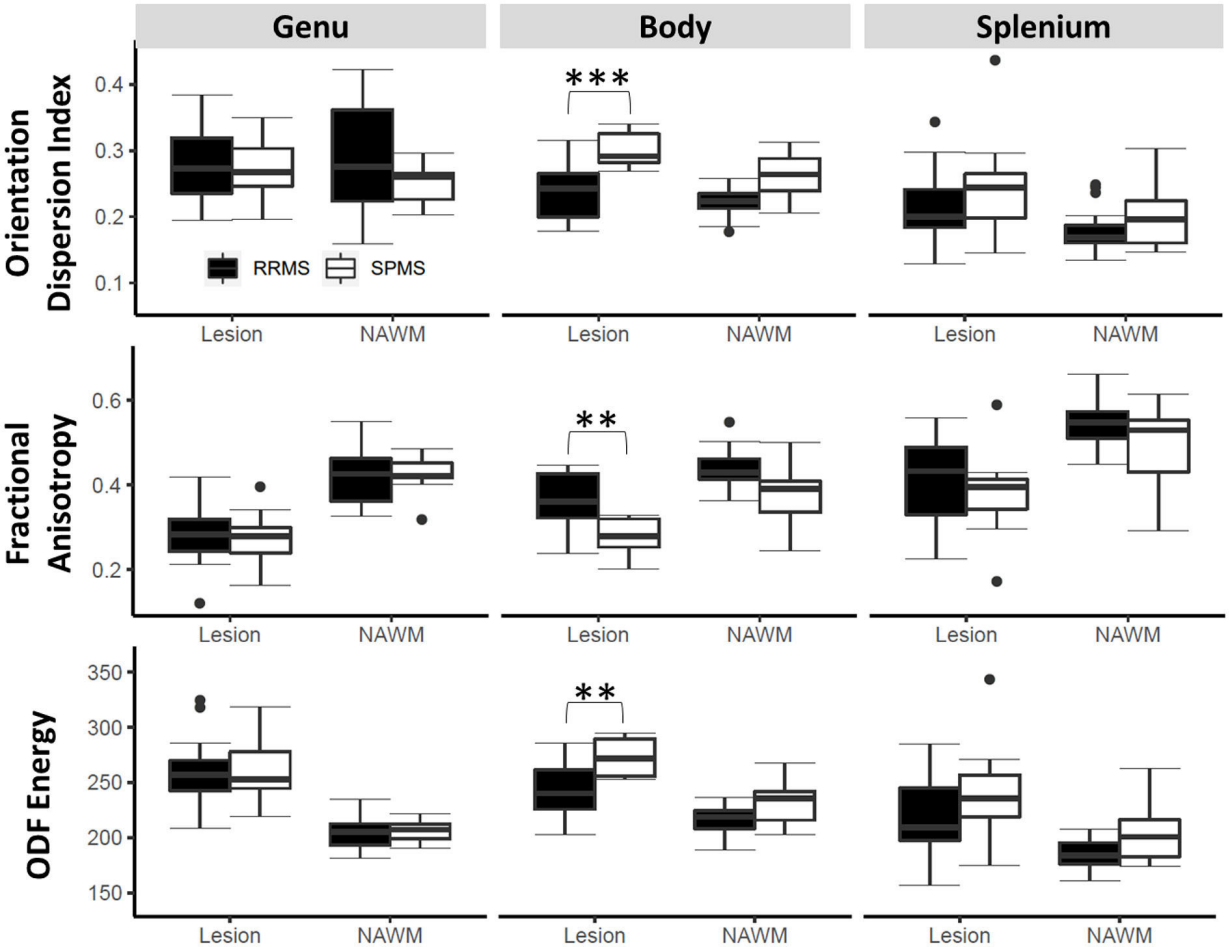
Tract ROI-based outcomes by cohort. Shown are findings in three diffusion measures on lesions and normal appearing white matter (NAWM) within three key regions of the corpus callosum. The stars indicate *post hoc* significance: *p < 0.05, **p < 0.01, ***p < 0.001. The boxes plot the median, interquartile range (IQR), and 1.5*IQR.

Following correction of multiple comparisons in ANOVA, three diffusion measures, namely, FA, ODF energy, and ODI detected differences between the cohorts (*p* < 0.0026) (Figure 5). All three measures showed significance in differentiating lesions (*p* < 0.001) and NAWM (*p* < 0.05) of the corpus callosum body. ODI and ODF energy were significant for the left (RRMS: 0.172, SPMS: 0.212) and right (RRMS: 207.64, SPMS: 240.49) optic radiations, respectively (*p* < 0.0026) following ANOVA. Pairwise comparisons highlighted ODI and ODF energy detecting cohort differences in the optic radiations for lesions (RRMS: 0.166, SPMS: 0.212, *p* < 0.01; RRMS: 212.15, SPMS: 238.15, *p* < 0.05) and NAWM (RRMS: 0.177, SPMS: 0.212, *p* < 0.05; RRMS: 203.13, SPMS: 242.83, *p* < 0.01).

### Along-tract statistics

**Figure 6:**
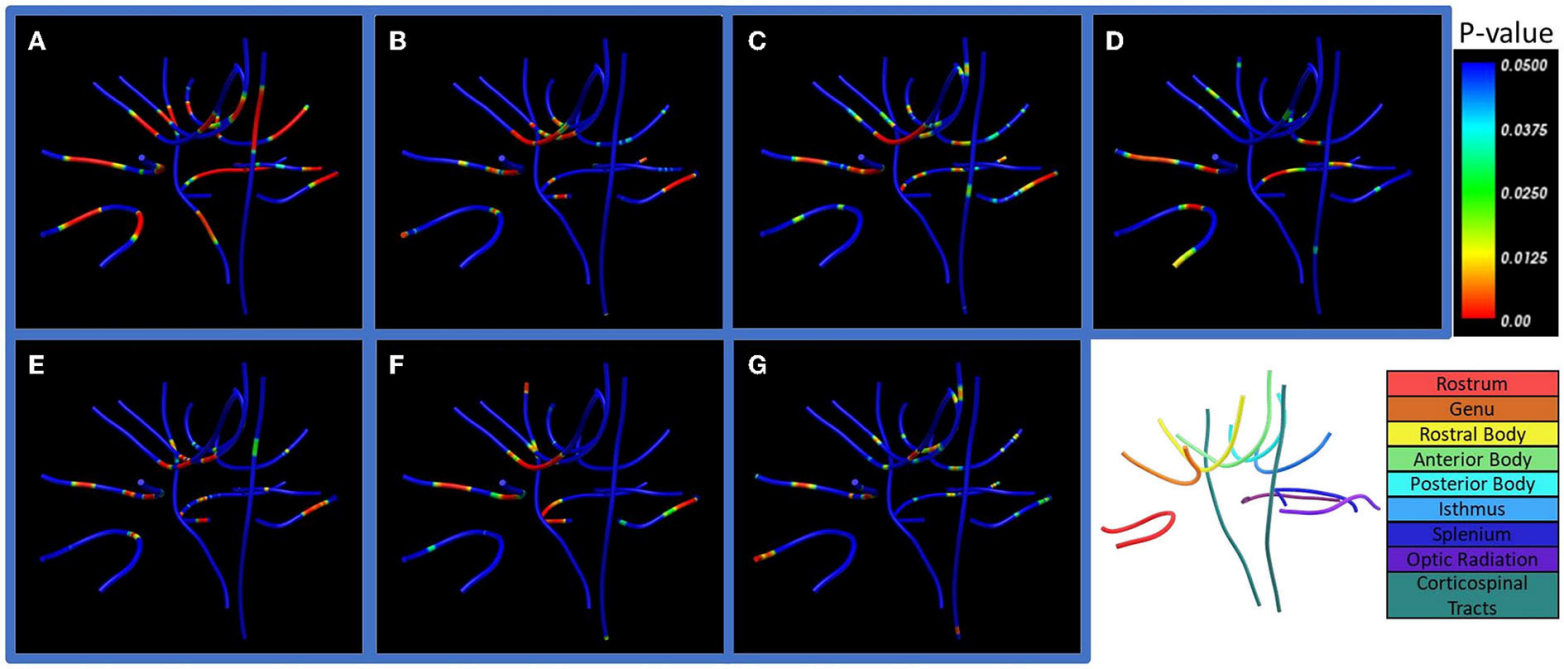
Along-tract statistics between cohorts. Shown are **(A)** lesion extent, **(B)** orientation dispersion index (ODI), **(C)** fractional anisotropy (FA), **(D)** apparent fiber density (AFD), **(E)** axonal diameter, **(F)** intracellular volume fraction (ICVF), and **(G)** orientation distribution function (ODF) energy. The *p*-value indicates significance of pointwise cohort differences prior to multiple comparison corrections. Bottom right: Labels of the seven corpus callosum segments, optic radiation, and corticospinal white matter tracts examined in the study.

There were prominent differences between cohorts in lesion extent when all tract values were averaged (Figure 6). With correction for multiple comparisons following ANOVA (*p* < 0.0024), lesion extent remained significant in the posterior body, isthmus, and splenium of the corpus callosum (*p* < 0.0024), and optic radiations in both hemispheres (*p* < 0.0001). T2 (16 mm) phase congruency was significant in the left but not in the right corticospinal tract (*p* < 0.0024). Lesion extent showed notable along-tract differences at major bihemispheric peaks appearing higher in patients with SPMS than in patients with RRMS; however, T2 (16 mm) phase congruency showed constant along-tract cohort differences. FA, ICVF, and ODI did not survive correction for multiple comparisons following ANOVA, but indicated whole-tract differences in the genu, rostral body, and both hemispheres of the optic radiation with significant along-tract differences in the callosal segments.

### Chronic lesion analysis

**Figure 7:**
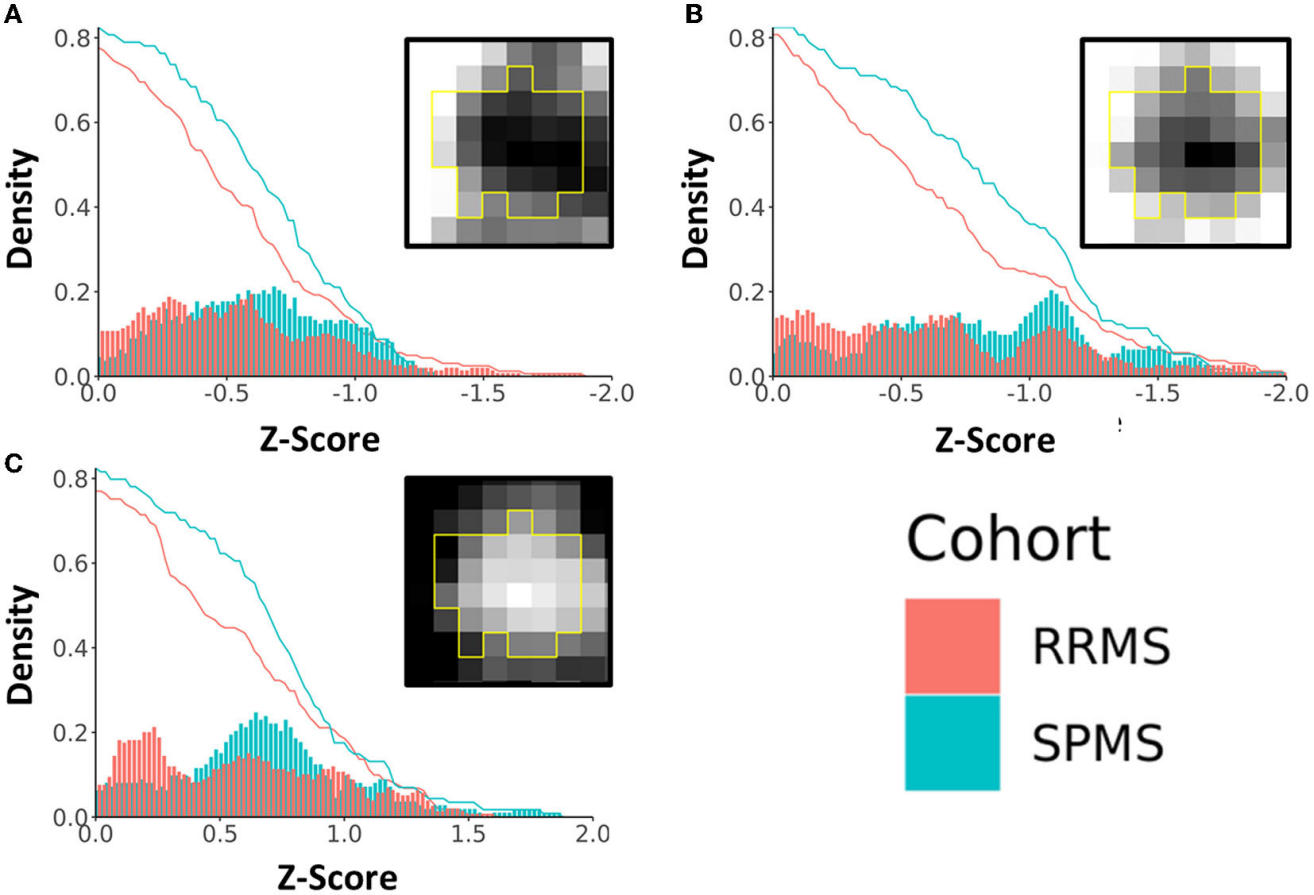
Density plot of chronic active lesions per cohort based on a common range of z-scores of diffusion metrics. Shown are results based on **(A)** fractional anisotropy (FA), **(B)** intracellular volume fraction (ICVF), and **(C)** axonal diameter. The histograms (bin size = 0.02) represent the percentage of chronic active lesions, and the red and blue curves represent the accumulated probability of the lesions with equal or more extreme z-scores. The boxed images show example lesion maps of the corresponding diffusion measures.

Lesions defined as chronic active had z-scores ranging from 0 to 2.0 (or 0 to −2.0 depending on the investigated measures) based on core vs. rim pathology analyses. Patients with SPMS showed 14.1, 18.1, and 13.2%, which corresponded to an average of 3.44, 3.84, and 3.33 more chronic active lesions in patients with SPMS than in patients with RRMS at z-scores between 0.5 and 1.5 according to axonal diameter, FA, and ICVF (Figure 7). In the 0.5 to 1.0 z-score range, the percentage of chronic active lesions in patients with SPMS was increased by 18.0%, 12.2%, and 4.9% according to axonal diameter, FA, and ICVF, respectively. This corresponded to an average of 3.28, 2.73, and 1.61 more chronic active lesions per SPMS patient than RRMS. According to axonal diameter, FA, and ICVF, 80–85% of the measured lesions were chronic active (>0, <0, <0, respectively); using AFD, ODF energy, and ODI, 40–65% of the measured lesions were chronic active (<0, >0, >0, respectively). Examining the number of chronic active lesions based on z-score thresholds, a 0.5 threshold showed cohort differences for all measures, and a threshold of 1.0 showed differences primarily with ICVF, which indicated an average lesion count of 4.56±1.67 for SPMS and 2.17±1.79 for RRMS (Table 2).

**Table 2:** The percentage and number of chronic active lesions in each cohort based on z-score definitions.

**Measures**		**RRMS**				**SPMS**			
		**>0.5**	**>1.0**	**0.5–1.0**	**0.5–1.5**	**>0.5**	**>1.0**	**0.5–1.0**	**0.5–1.5**
Diameter	% of total	45.3	18.6	26.7	44.7	62.3	17.5	44.7	58.8
	#_lesion_/pt Mean (s.d.)	4.06 (3.19)	1.67 (1.24)	2.39 (2.45)	4.00 (3.12)	7.89 (2.20)	2.22 (1.56)	5.67 (1.58)	7.44 (1.94)
FA	% of total	44.1	12.4	31.7	41.6	59.6	15.8	43.9	59.7
	#_lesion_/pt Mean (s.d.)	3.94 (3.33)	1.11 (1.23)	2.83 (2.60)	3.72 (3.23)	7.56 (2.92)	2.00 (1.41)	5.56 (2.92)	7.56 (2.92)
ICVF	% of total	50.9	24.2	26.7	44.7	67.5	36.0	31.6	57.9
	#_lesion_/pt Mean (s.d.)	4.56 (3.33)	2.17 (1.79)	2.39 (2.23)	4.00 (3.18)	8.56 (3.00)	4.56 (1.67)	4.00 (2.12)	7.33 (2.78)

## Discussion

Through advanced analysis of diffusion and anatomical brain MRI, we have detected significant differences between RRMS and SPMS participants in different scales of tissue pathology. The SPMS individuals show greater NAWM damage across nearly all diffusion and phase congruency-based texture measures of the whole brain. Similarly, increased tissue damage in SPMS is also manifested in both lesions and NAWM within two of the three critical brain white matter tracks as detected by orientation-informed FA, ODF energy, and ODI diffusion measures. Furthermore, along-tract statistics highlighted significant differences in lesion extent within several callosal segments among others. This is accompanied by dramatically increased percentage and number of chronic active lesions in SPMS compared with RRMS subjects.

It is well known that NAWM plays an important role in disease progression in MS (De Santis et al., 2017; Vavasour et al., 2017). However, the exact patterns of change during the process are unclear. Our findings indicate that there is increased tissue damage in the NAWM of SPMS at both micro- and macroscopic levels as shown by advanced diffusion MRI and phase congruency measures. Furthermore, the damage may vary by brain region or tissue type. The observation that the SPMS NAWM shows lower ICVF at p95, greater axonal diameter at p50, and greater orientation dispersion index at p50 histogram regions than RRMS suggests that high-density white matter bundles with small diameter and low dispersion are most susceptible to NAWM damage. Texture measures also detected significant differences between RMS and SPMS primarily at sharper structure transition points, as reflected by high phase congruency and low weighted mean phase values. The increase in FLAIR phase congruency and the decrease in weighted mean phase may reflect an increased variation in local tissue structure resulting from loss of homogenous myelination. In contrast, the decreased phase congruency and increased weighted mean phase of T2-w MRI may reflect increased signal homogeneity from corticospinal fluid and inflammation, which are suppressed in FLAIR. Pinning down the specific patterns of difference in pathology between RRMS and SPMS would permit targeted analysis of brain NAWM, thereby increasing the efficiency in the search of non-lesion mechanisms of disease progression in MS.

In contrast to whole-brain NAWM analysis, tract-based ROI analysis focused on major white matter tracts known to impact patient function (Reich et al., 2008, 2009; Llufriu et al., 2012). The corpus callosum plays a significant role in interhemispheric communication. Therefore, it is not surprising to observe significant increases in diffusion damage in the body of corpus callosum of SPMS compared with RRMS in both the NAWM and lesion areas. Cohort differences in the corticospinal tract were also detected by a few diffusion measures showing worsening in SPMS than RRMS but were unilateral and focused on lesions only within the tract. These findings indicate the severity of tissue damage in critical brain regions of SPMS, as all the associated regions of the white matter tracts are important regulators of motor functions (Sechi et al., 2019). Furthermore, current evidence may also highlight that lesion damage within major white matter tracks remain to be critical contributors of advanced disease in MS (Martínez-Heras et al., 2020).

Along-tract analysis offered an opportunity to analyze tissue structure properties along the entire length of white matter tracts. Lesion extent appears to be the most significant measure that differentiates SPMS from RRMS, showing increased quantity in patients with SPMS in all the tracts detected, especially the posterior regions of the corpus callosum and optic radiation. Lesion extent herein measures the percentage of image voxels belonging to lesion areas vs. NAWM. While the findings again highlight the critical role of lesions, consistent with results from ROI-based tract analysis above, lesion extent provides a different measure of pathology in the context of tracts. Lesion extent showed significant cohort differences at symmetric regions between hemispheres, presenting with greater lesion burden than adjacent regions for all tracts. The symmetrical changes in lesion extent between hemispheres are different from the changes in FA, ICVF, and ODI, which showed cohort differences mainly in the midsagittal regions of the brain, such as the genu and rostral body of corpus callosum, which warrantee further investigation.

Taking advantage of the sensitivity of diffusion MRI measures to microstructural changes, we have also investigated the activity of chronic MS lesions through the core-rim framework. The dichotomy of the lesion core and rim has been investigated in chronic active lesions previously by others to demonstrate DTI sensitivity to regional differences (Klistorner et al., 2018, 2021). In this study, we expanded the lesion core and rim examinations through the z-score framework across a range of diffusion microstructure measures, allowing detailed understanding of individual lesions in both RRMS and SPMS. In this study, cohort differences in the distribution of chronic active lesions over the 0.5–1.5 range of z-scores may highlight a critical threshold territory useful for identifying progression from RRMS to SPMS. A z-score of 0.5 indicates a reasonable degree of pathological differences between the core and rim, which may serve as an appropriate threshold to define chronic-active lesions, deserving further verification. ODI, ODF energy, and AFD did not show clear core-rim differences. This may result from their dependence on diffusion orientation models, which may be influenced by reductions in axonal density resulting from pathological damage (Schneider et al., 2017).

There are some limitations in this study. The sample size is small that may limit generalization of our findings. But significant differences were found in various measures between RRMS and SPMS. In addition, half of our diffusion data are derived from prediction based on the available diffusion MRI in HARDI analysis. While this approach is subject to further confirmation, our pilot results using predicted data demonstrate validity (Murray et al., 2022), and such an approach can be extremely beneficial to clinical scenarios where imaging acquisition time is limited. Furthermore, our diffusion measures present with similar patterns to those shown in the literature, and prior research has found that the outcome measures are equivalent between single-shell and multi-shell HARDI (Oladosu et al., 2021). Another limitation is the use of two different datasets. Nonetheless, the impact of dataset combination appears to be mitigated by the similarity of their acquisition protocols, our use of tested techniques to harmonize datasets, and our integration of robust image preprocessing strategies. Specifically, the difference of b values between the two datasets used in our study is 150 s/mm^2^ which is far from the allowed threshold (500 and 1,500 s/mm^2^) in performing harmonization of diffusion MRI (Cetin Karayumak et al., 2019). Furthermore, our quantitative results on both the variance and SNR of ROIs in brain white matter confirm the feasibility of our harmonization approaches. Similarly, our use of the contrast-limited adaptive histogram equalization method in phase congruency-based texture analysis should have also helped minimize the impact of corresponding protocol differences. In the future, we seek to validate our findings using additional datasets, extend the z-score paradigm for chronic active lesion analysis to images with different resolutions and with smaller lesions, and investigate the relationship between chronic active lesion activity and patient function in MS with or without progression.

In summary, using advanced diffusion MRI and image texture analysis methods, we found significant differences between RRMS and SPMS subjects across a wide range of measures of brain microstructure. The SPMS participants appear to have increased NAWM pathology at both microscopic and macroscopic degrees compared with RRMS participants. Moreover, lesion pathology seems to still play a critical role in disease development in MS, as highlighted by both within-tract and along-tract analyses. Furthermore, using advanced diffusion MRI measures, this study has also developed a novel method for defining the activity of chronic active lesions, a much-needed dimension in understanding functional decline in MS. Overall, this study may provide a useful foundation for future studies of disease progression in MS, as represented by joint analysis of different scales of tissue pathology.

## Data availability statement

The original contributions presented in the study are included in the article/Supplementary material, further inquiries can be directed to the corresponding author.

## Ethics statement

The studies involving human participants were reviewed and approved by the Conjoint Health Research Ethics Board, University of Calgary. The patients/participants provided their written informed consent to participate in this study.

## Author contributions

OO participated in study design, data analysis and interpretation, and manuscript draft and edit. W-QL participated in study design, data acquisition and analysis, and manuscript edit. LB, BP, and LM participated in study design, data acquisition, and manuscript edit. YZ participated in study design, data acquisition and interpretation, and manuscript draft and edit. All authors contributed to the article and approved the submitted version.

## Funding

This study was supported by the Alberta Innovates and Multiple Sclerosis Society of Canada for acquiring study resources, operating the study, and disseminating results. This research was also benefited from personnel awards from the Alberta Innovates and Canadian Network for MS Clinics (W-QL) and scholarship support from the NSERC CREATE I3T program, Bonvicini Neuroscience Scholarship, the Government of Alberta Queen Elizabeth II Graduate Scholarship, and Alberta Graduate Education Scholarship (OO).

## Conflict of interest

The authors declare that the research was conducted in the absence of any commercial or financial relationships that could be construed as a potential conflict of interest.

## Publisher's note

All claims expressed in this article are solely those of the authors and do not necessarily represent those of their affiliated organizations, or those of the publisher, the editors and the reviewers. Any product that may be evaluated in this article, or claim that may be made by its manufacturer, is not guaranteed or endorsed by the publisher.
