# Supplementary material for: Advanced diffusion MRI and image texture analysis detect widespread brain structural differences between relapsing-remitting and secondary progressive multiple sclerosis
Source: Front Hum Neurosci. 2022 Aug 12;16:944908. doi: 10.3389/fnhum.2022.944908 (PMC9413838; doi:10.3389/fnhum.2022.944908)
Supplement: Supplementary file 1 [file Table_1.DOCX]

**Supplementary Table 1**: The mean (standard deviation) variance and signal-to-noise ratio of white matter regions of interest in RRMS participants from Dataset1 and Dataset2.


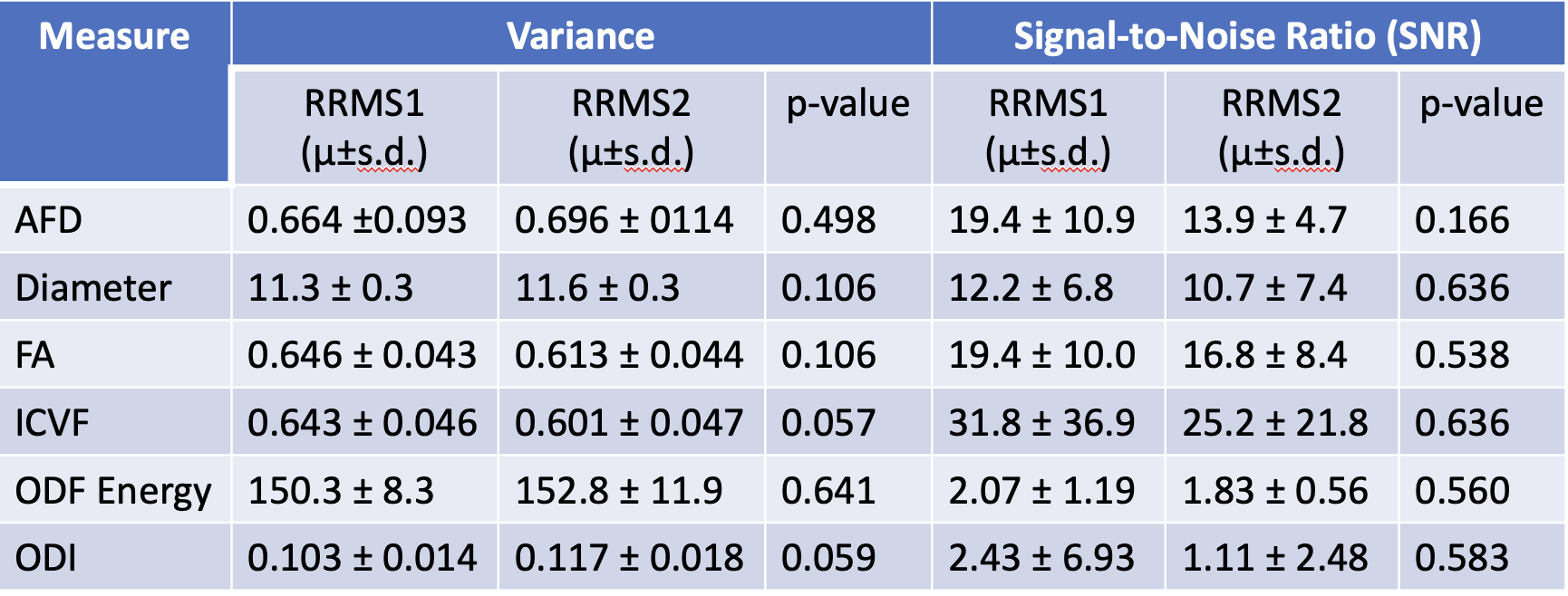


Note: AFD: apparent fiber density; FA: fractional anisotropy; ICVF: intracellular volume fraction; ODF: orientation density function; ODI: orientation dispersion index.
